# Impact of migratory flows and socio-environmental factors on dengue epidemiology in Oaxaca, Mexico

**DOI:** 10.3389/fsoc.2025.1617789

**Published:** 2025-07-09

**Authors:** Diana Matías-Pérez, Araceli Guerra-Martínez, Emilio Hernández-Bautista, Alma Dolores Pérez-Santiago, Alma Lilia Antonio-Cruz, Iván Antonio García-Montalvo

**Affiliations:** División de Estudios de Posgrado e Investigación, Tecnológico Nacional de México, Instituto Tecnológico de Oaxaca, Oaxaca, Mexico

**Keywords:** dengue, migrants, public health, dynamics of migration, Oaxaca

## Abstract

Oaxaca, state rich in culture and biodiversity, is currently facing a growing challenge due to the combined effects of mass migration and a significant increase in dengue cases. In recent years, the continuous influx of migrants seeking better opportunities has transformed the region’s social and economic landscape, with a severe impact on public health. Dengue, transmitted by *Aedes mosquitoes*, has become a critical concern for health authorities, particularly given the favorable climatic conditions for vector proliferation in Oaxaca. The arrival of thousands of migrants, many from countries with endemic dengue outbreaks, has facilitated the introduction of new virus serotypes into the region. Their precarious conditions during their transit, including makeshift shelters with few sanitary facilities, create environments conducive to mosquito proliferation. This is aggravated by the accumulation of garbage and lack of access to potable water. The public health response is urgent and multifaceted, with educational campaigns on dengue prevention targeting both residents and migrants. However, these initiatives face the challenge of reaching a diverse population with different levels of information and access to essential services. Collaboration between non-governmental organizations and government is necessary to address the immediate public health needs of these migratory flows.

## Introduction

Oaxaca, a state renowned for its cultural and biological diversity, is currently confronting a dual challenge: mass migration and a sharp increase in dengue fever cases. In recent years, the region has experienced a substantial rise in the number of migrants crossing its territory in search of improved living conditions (Sánchez-Montijano and Zedillo-Ortega, 2022). Oaxaca is one of the most ethnically and culturally diverse states in Mexico, with an estimated population of 4.1 million inhabitants distributed in 570 municipalities, which represents the highest municipal fragmentation in the country (Instituto Nacional de Estadística y Geografía (INEGI), 2020). The population is mainly concentrated in the capital, Oaxaca de Juárez, and in the regions of the Central Valleys, the Coast, and the Isthmus of Tehuantepec. However, there are marked socioeconomic differences and differences in access to health services between urban and rural regions, as well as between indigenous and mestizo communities (Consejo Nacional de Evaluación de la Política de Desarrollo Social (CONEVAL), 2023).

These inequalities have a direct impact on the population’s vulnerability to vector-borne diseases such as dengue. Since the 1990s, Oaxaca has experienced fluctuating dengue incidence, with sporadic outbreaks. In recent years, however, the incidence has increased steadily, a trend attributed to both climatic and social factors—including urbanization, migration, and the emergence of new viral serotypes (SSO, 2023; Zubieta-Zavala et al., 2018). This constant flow of people has not only transformed Oaxaca’s social and economic landscape but has also had a profound impact on public health, particularly in the spread of dengue fever (Zubieta-Zavala et al., 2018).

Dengue, a viral disease transmitted by *Aedes* mosquitoes, has become a critical concern for health authorities. Oaxaca’s climatic conditions, characterized by intense rainfall and warm temperatures, create an environment conducive to reproducing these vectors (Liu et al., 2023). The impact of migratory flows has added a layer of complexity to this situation. The arrival of thousands of migrants, many of whom come from countries with endemic dengue outbreaks, has facilitated the introduction of new virus serotypes into the region (Rodriguez-Morales et al., 2024).

The precarious conditions in which many migrants live during their transit are a determining factor in the spread of dengue fever. In makeshift shelters and centers of migratory mobility, where sanitary facilities are insufficient, and hygiene is deficient, ideal breeding grounds for the proliferation of the *Aedes aegypti* mosquito are generated (Massaro et al., 2019). The accumulation of garbage and lack of access to potable water further aggravate this situation. With each new group of migrants arriving in Oaxaca, the risk of contagion multiplies, creating a vicious cycle that threatens both the migrants and the local population (Jasso-Vargas and Cejudo-Espinosa, 2021).

The health response to this problem is urgent and multifaceted. Authorities have begun implementing educational campaigns on dengue prevention aimed at residents and migrants. However, these initiatives face the challenge of reaching a diverse population with different levels of information and access to essential services (Estallo et al., 2024). Collaboration between non-governmental organizations and government is essential to address the immediate public health needs arising from migratory crossings (Hossain et al., 2024).

The pressure on local health systems intensifies as the number of positive cases continues to rise in Oaxaca. Hospitals and clinics are seeing increased occupancy due to the care required by dengue patients, putting the system’s ability to handle other medical emergencies at risk (Ríos-Bracamontes et al., 2024, PAHO, 2023, 2024, 2025). In this context, it is crucial to understand that the migratory phenomenon is not only a humanitarian challenge but also a determining factor in public health that requires immediate attention (Bozorgmehr et al., 2023).

The intersection between migration and public health in Oaxaca is a microcosm of broader global problems. Addressing this crisis requires effective policies to control dengue and a deep understanding of the social and economic dynamics that drive migration (Masferrer and Pedroza, 2022). Only a comprehensive approach that considers health and humanitarian needs can mitigate the impact of dengue in this diverse and vulnerable region. Globally, the movement of populations has been linked to the spread of vector-borne diseases such as dengue (Bozorgmehr et al., 2023). In Mexico, dengue incidence has increased in recent years, with Oaxaca experiencing one of the most significant surges (SSO, 2023). The objective is to identify trends, gaps, and the scope of existing research on the interaction between migration, climatic conditions, serotype circulation, and socioeconomic factors in dengue transmission in the region.

## Methodology

### Research design

This study employs a systematic literature review approach to analyze the relationship between migration dynamics and dengue risk in Oaxaca, Mexico. The objective is to identify trends, gaps, and the scope of existing research on the interaction between migration, climatic conditions, serotype circulation, and socioeconomic factors in dengue transmission in the region.

### Literature search strategy

An exhaustive search was conducted in relevant academic databases, including Scopus, Web of Science, PubMed, and Google Scholar. The search terms used included: “dengue in Oaxaca,” “migration and public health,” “dengue serotypes,” “climate change and dengue,” and “migration dynamics in Mexico.” Original articles, reviews, technical reports from health agencies, and gray literature relevant to the regional context were considered.

### Inclusion and exclusion criteria

We included peer-reviewed studies, official reports, and academic literature published between 2015 and 2025 that addressed the relationship between migration, dengue, and associated factors in Oaxaca. Both qualitative and quantitative research, as well as epidemiological reports and public policy documents, were considered. Non-academic sources, journalistic notes, studies that focused solely on the clinical aspects of dengue without links to migration or social factors, and documents not available in Spanish or English were excluded.

### Data extraction

Initial screening was performed by reviewing titles and abstracts, followed by a full-text evaluation of the pre-selected papers. A standardized format was used to extract relevant information, including authors, year of publication, study objectives, sample characteristics or context, methodology, main findings, and gaps identified in the literature.

### Analysis

Descriptive statistics were used to summarize the number and type of studies focused on each aspect (migration, climate, serotypes, infrastructure). A thematic analysis was conducted to identify recurring patterns and emerging trends in the literature. Findings were synthesized to highlight how migration and other non-climatic factors have influenced dengue dynamics in Oaxaca, as well as areas where evidence is limited or contradictory.

### Presentation of results

The results are presented in a descriptive summary, complemented by a Table illustrating the main internal and external migration flows in Oaxaca. The thematic synthesis presents the main findings, identifies gaps in the literature, and provides a basis for policy recommendations and future research.

## Results and discussion

A systematic literature review was conducted in the Scopus, Web of Science, PubMed, and Google Scholar databases. Publications from 2015 to 2025 on migration, dengue, and associated factors in Oaxaca were included in this review. After filtering titles, abstracts, and full-text evaluation, 27 relevant documents were selected, including original articles, reviews, technical reports, and gray literature (see Table 1). This database enabled us to identify trends, gaps, and emerging patterns in the influence of migration and socio-environmental factors on the epidemiology of dengue in the region.

**Table 1:** Selected references on migration, dengue and associated factors in Oaxaca and Mexico (2015–2025).

No.	Reference (Author, year)	Type of document	Main objective
1	Zubieta-Zavala et al. (2018)	Original article	To analyze the incidence of dengue in Mexico and associated social factors.
2	SSO (2023)	Technical Report	Report cases and circulating serotypes of dengue in Oaxaca.
3	Rodriguez-Morales et al. (2024)	Original article	Introduction of dengue serotypes by migrants.
4	Instituto Nacional de Estadística y Geografía (INEGI) (2020)	Technical report	Demographic and migratory statistics of Oaxaca.
5	Consejo Nacional de Evaluación de la Política de Desarrollo Social (CONEVAL) (2023)	Technical report	Social inequality and access to services in Oaxaca.
6	SSO (2023)	Technical report	Annual analysis of dengue cases and migration.
7	Secretaría de Salud de Oaxaca (SSO) (2021)	Technical report	Dengue control strategies in Oaxaca.
8	PAHO (2023)	Technical report	Epidemiological situation of dengue in the Americas.
9	PAHO (2024)	Technical report	Update on outbreaks and serotypes in Mexico.
10	PAHO (2025)	Technical report	Projections and recommendations for dengue control.
11	Mendoza-Cano et al. (2025)	Original article	Adaptability of *Aedes aegypti* in urban environments.
12	Wilke et al. (2020)	Original article	Risk factors for dengue fever in urban areas.
13	Liu et al. (2023)	Review article	Impact of climate change on vector-borne diseases.
14	Massaro et al. (2019)	Original article	Sanitary conditions in migrant shelters and risk of dengue fever.
15	Jasso-Vargas and Cejudo-Espinosa (2021)	Original article	Socio-environmental factors in dengue transmission.
16	Estallo et al. (2024)	Review article	Strategies for dengue prevention in migratory contexts.
17	Hossain et al. (2024)	Original article	Interinstitutional collaboration in public health in the face of migration.
18	Sánchez-Montijano and Zedillo-Ortega (2022)	Review article	Recent migration flows in southern Mexico.
19	Roldán-Barrios (2024)	Original note	Circulation of dengue serotypes in Oaxaca.
20	Dzul-Manzanilla et al. (2021)	Review article	Spatial review that includes southern states such as Oaxaca, for the identification of urban foci of arbovirus transmission, supporting targeted vector control strategies.
21	Gutierrez et al. (2023)	Original article	Genomic and epidemiological study analyzing the simultaneous circulation of dengue and chikungunya in Mexico, with relevant data for southern and southeastern regions, including Oaxaca.
22	Vargas-Navarro et al. (2021)	Review article	Systematic review that addresses clinical and epidemiological aspects and challenges in the control of dengue fever in Mexico, with mention to endemic regions such as Oaxaca.
23	Ríos-Bracamontes et al. (2024)	Original article	Impact of increased dengue fever on health systems.
24	Bozorgmehr et al. (2023)	Review article	Migration and emerging infectious diseases.
25	Masferrer and Pedroza (2022)	Original article	Public policies on migration and health.
26	Sirisena et al. (2021)	Original article	Coinfection with several serotypes increases the risk of severe dengue.
27	Butterworth et al. (2017)	Original article	Relationship between climate and *Aedes aegypti* proliferation.

### Climate change, dengue vector adaptability and migration

Climate change, the adaptability of the dengue vector, and the migratory flow in Oaxaca are interrelated in a cycle that aggravates the public health crisis in the region. Rising temperatures, driven by global warming and phenomena like El Niño, have created ideal conditions for the proliferation of the *Aedes aegypti* mosquito, the primary transmitter of dengue. This mosquito reproduces more rapidly in hot and humid climates, increasing the risk of dengue outbreaks in communities with temperatures exceeding 25°C (Butterworth et al., 2017). Oaxaca has a predominantly warm, sub-humid climate, with average annual temperatures ranging from 22°C to 28°C and a rainy season that extends from May to October. These conditions favor the reproduction of the *Aedes aegypti* mosquito, the primary vector of dengue.

Migration flows in Oaxaca have increased significantly, with migrants from Venezuela, Haiti, and several other Central American countries. Ongoing migration to Oaxaca, driven by economic and social factors, has added another layer of complexity. Migrants, often in precarious conditions and with limited access to essential services, create environments conducive to mosquito breeding. The accumulation of waste and stagnant water in temporary settlements facilitates the spread of dengue fever. In addition, new population groups can introduce different virus serotypes, increasing the risk of severe infections (Cruces et al., 2023). Dengue serotypes not previously seen in the region have been introduced. This is of concern because co-infection with different serotypes can lead to more severe forms of the disease. Simultaneous circulation of several serotypes increases the likelihood of severe complications and hospitalization (Sirisena et al., 2021).

In 2020, Oaxaca reported 1,095 confirmed cases of dengue, with a case fatality rate of 3.19%. During 2021, the situation stabilized with 1,095 cases, but in 2022, a significant increase was observed, reaching 5,190 probable cases and 11,695 confirmed cases. This increase was associated with the circulation of several virus serotypes and favorable climatic conditions for the proliferation of the *Aedes aegypti* mosquito. In 2023, the crisis intensified further. As of epidemiological week, number 34 in August, 709 confirmed cases were reported, with a breakdown that included 338 cases of non-severe dengue, 345 with alarm signs, and 26 severe cases. Compared to last year’s period, this represented an alarming 500% increase in dengue cases. In addition, three deaths related to the disease were recorded (SSO, 2023).

Specific groups, such as young children and older people, are more susceptible to severe dengue complications, increasing the burden on local health systems despite efforts with frequent spraying campaigns. The adaptability of *Aedes aegypti* is remarkable; this mosquito not only adapts to diverse climatic conditions but can also survive in densely populated urban environments. This means that areas affected by the migratory flow are particularly vulnerable to dengue outbreaks, especially when climatic conditions favor the vector (Wilke et al., 2020; Mendoza-Cano et al., 2025). The combination of these factors underscores the urgent need to implement comprehensive strategies that address both dengue control and the socioeconomic conditions of migrants in Oaxaca. Over the last five years, the simultaneous circulation of the four dengue virus serotypes (DENV-1, DENV-2, DENV-3, and DENV-4) has been documented in Oaxaca, with a predominance of DENV-2, and the recent introduction of DENV-3 has been associated with migratory flows (see Table 2) (Roldán-Barrios, 2024; SSO, 2023; PAHO, 2023; PAHO, 2024; PAHO, 2025).

**Table 2:** Confirmed cases of dengue in Mexico and Oaxaca, migration flow from Central America and prevalent serotypes (2023–2025) (PAHO, 2023, 2024, 2025; Mendoza-Cano et al., 2025).

Year	Confirmed cases Mexico	Confirmed cases Oaxaca	Migratory flows from Central America to Mexico and Oaxaca	Prevalent serotype
2023	22, 950 cases	709 cases	Substantial increase in irregular migration (+62% in the first 8 months of 2023 vs. 2022); Mexico receives a large flow, while Oaxaca has a smaller presence; the leading countries of origin are Guatemala, Honduras, and El Salvador.	predominant DENV-1 and DENV-2
2024	56, 548 cases	3, 314 cases	Irregular migration almost triples in the first half of 2024 compared to 2023; an intense flow is observed at the southern border (Chiapas), while Oaxaca experiences reinforced surveillance in coastal areas.	DENV-2 and DENV-3, with an increase of DENV-4 in some regions
2025	1, 781 cases (up to week 8)	20 cases (up to week 19)	The irregular migration flow remains high, with 712,226 events reported in the first half of 2025; Oaxaca continues to have a low number of initial cases.	DENV-2 and DENV-3 continue to predominate

### Human mobility as a catalyst for the spread of dengue in Oaxaca

Migratory caravans play a crucial role in the spread of diseases, such as dengue fever, in Oaxaca. This phenomenon has intensified in recent years, coinciding with an alarming increase in dengue cases in the state. According to Dr. Ignacio Zárate Blas, Oaxaca’s Director of Prevention and Health Promotion, the arrival of migrants has introduced new serotypes of the dengue virus, especially serotype 3, which had not previously been observed in the region. The constant flow of migrants through Oaxaca, a key point on the routes to the United States, has facilitated the circulation of different dengue serotypes (Roldán-Barrios, 2024; Dzul-Manzanilla et al., 2021).

Historically, the state had maintained a prevalence of serotype 2, but with migration from South American countries where dengue is endemic, such as Brazil and Colombia, co-circulation of up to four serotypes has been observed in several municipalities (Roldán-Barrios, 2024). This viral diversity significantly complicates the health situation, as the immune system of people exposed to one serotype may react more severely when encountering another. To address these challenges, the State Government has established a health care system for migrants. During the transit of the caravans, medical consultations and emergency services have been provided in different localities (CCS, 2024). In the San Miguel Suchixtepec and Santo Tomás Tamazulapam municipalities, multiple consultations were carried out without reporting cases that met the operational definition of diseases subject to epidemiological surveillance. The authorities have implemented preventive measures through vector control and health promotion brigades.

These include the installation of filters for the early detection of probable cases of dengue and malaria, as well as educational campaigns on the importance of hand washing and adequate hydration to prevent diarrheal diseases. Antivectorial activities have also been carried out to eliminate mosquito breeding sites and promote hygienic practices among local communities and migrants. Despite these efforts, significant challenges remain (SSO, 2023). The scarcity of medical resources and inadequate infrastructure in the most affected areas complicate the health response. Authorities must coordinate with local and national organizations to ensure that migrants receive timely medical care without fear of being separated from their family groups.

### Non-climate trends related to population growth, urbanization, and commerce

In recent decades, Oaxaca has experienced sustained and significant demographic growth driven by the increase in natural population and internal migratory movements. This population growth is mainly concentrated in urban and peri-urban areas, where accelerated expansion has generated a higher density of inhabitants in increasingly smaller spaces (Varela-Llamas et al., 2017). This population concentration creates a favorable scenario for the transmission of dengue fever since the proximity between people facilitates contact with the mosquito vector and, therefore, the spread of the virus.

In many Oaxacan communities, infrastructure and public services have failed to expand and modernize at the pace necessary to meet these demands. This gap between population growth and infrastructure development creates environmental conditions that favor the proliferation of the dengue-transmitting mosquito. Poor solid waste management in urban and peri-urban areas, where garbage accumulates without efficient collection, generates multiple pockets of stagnant water in waste, tires, cans, and other objects, which serve as perfect habitats for mosquito breeding (Krystosik et al., 2020).

In rural and semi-urban areas of Oaxaca, population growth has also driven the expansion of informal settlements and marginalized neighborhoods, often located on irregular or inaccessible land. These areas present precarious sanitary conditions, lacking basic infrastructure such as drainage, public lighting, and access to drinking water. Urbanization implies profound transformations in land use, where extensive areas previously destined for natural vegetation, green spaces, or agricultural activities are converted into residential, commercial, and industrial zones.

This process of landscape change not only modifies the physical environment but also negatively impacts local ecosystems and biodiversity. The reduction of natural areas and the alteration of habitats affect the presence and abundance of natural predators that regulate *Aedes aegypti* mosquito populations, thus facilitating the proliferation of this vector (Diaconu et al., 2025; Aguilar et al., 2022; Gutierrez et al., 2023; Vargas-Navarro et al., 2021). In the specific case of Oaxaca, internal migration from rural communities to urban centers, motivated mainly by the search for better job, educational, and service opportunities, has caused a notable increase in population density in cities such as Oaxaca de Juarez.

Trade and mobility of people and goods are key non-climatic trends that significantly influence dengue transmission dynamics in Oaxaca. Increasing economic and social integration within the state and with other regions of the country facilitates the constant circulation of individuals who may be asymptomatic or symptomatic carriers of the virus, as well as the transport of objects and materials that may harbor mosquito eggs or larvae. The transportation of goods, particularly when water containers are used for the storage or transport of products, represents an indirect vector for the spread of the mosquito. These conditions allow the immature stages of the mosquito to survive and move to new areas, thus expanding its geographical range. Similarly, human mobility due to work, commercial, or tourist activities contributes to introducing and reintroducing the virus in different localities, facilitating the appearance of epidemic outbreaks in previously free or low-incidence areas (Coalson et al., 2021).

In Oaxaca, local and regional trade connects urban centers with dispersed rural and indigenous communities. This dynamic commercial network increases contact between diverse populations and the vector, enhancing the spread of dengue across different geographic locations. However, the absence of specific and targeted prevention campaigns in strategic sites related to trade and transportation limits the effectiveness of control actions, making comprehensive disease management difficult. In addition to international migration, Oaxaca experiences significant internal migration, mainly from rural communities to urban centers such as Oaxaca de Juárez and Salina Cruz, in search of better job and educational opportunities (Varela-Llamas et al., 2017). This phenomenon contributes to population concentration and the expansion of informal urban areas, increasing the risk of dengue transmission.

### Recommended strategies for dengue mitigation in migratory settings

Given the complex interaction between migration, climatic conditions, and dengue transmission in Oaxaca, the following comprehensive strategies are proposed:

#### Strengthening epidemiological surveillance

Implement real-time monitoring systems for the early detection of outbreaks and the identification of circulating serotypes, particularly in areas with high population mobility.

#### Improve sanitation infrastructure

Prioritize access to safe drinking water and adequate waste management in temporary shelters and communities receiving migrants.

#### Multilingual educational campaigns

Develop prevention materials in Spanish and indigenous languages, targeting both residents and migrants, to eliminate breeding sites and raise awareness of warning signs.

#### Inter-institutional coordination

Promote collaboration among health authorities, migration agencies, civil society organizations, and international organizations to ensure timely medical attention without discrimination.

#### Targeted vector control

Intensify fumigation, larval control, and distribution of mosquito nets in areas identified as high risk due to the concentration of migrants and previous outbreaks.

## Conclusion

In conclusion, the convergence of factors such as increased migratory flows, climate change, accelerated urbanization, and social inequalities has generated a critical scenario for public health in Oaxaca, as evidenced by the alarming increase in dengue cases and the introduction of new viral serotypes. The massive arrival of migrants, many of them from endemic regions, has facilitated the circulation of serotypes not previously present in the state, increasing the risk of serious infections and complicating the health response. The precarious conditions in which migrants travel and reside favor the proliferation of the *Aedes aegypti* vector and, consequently, the transmission of dengue among both migrants and the local population. This is compounded by the vulnerability of specific groups, such as children and older people, as well as the growing pressure on local health systems, which face resource and infrastructure limitations. Although the authorities have implemented educational campaigns, vector control measures, and targeted medical care, significant challenges persist in reaching a diverse and constantly mobile population. It is essential to strengthen inter-institutional coordination and collaboration between governmental and non-governmental agencies to ensure a comprehensive, timely, and culturally relevant response.
